# Olive Tree Biophenols in Inflammatory Bowel Disease: When Bitter is Better

**DOI:** 10.3390/ijms20061390

**Published:** 2019-03-20

**Authors:** Tiziana Larussa, Maria Imeneo, Francesco Luzza

**Affiliations:** Department of Health Sciences, University of Catanzaro “Magna Graecia”, Viale Europa, 88100 Catanzaro, Italy; tiziana.larussa@gmail.com (T.L.); graziaimeneo@hotmail.it (M.I.)

**Keywords:** inflammatory bowel disease, ulcerative colitis, Crohn’s disease, olive oil, olive leaves, biophenols, oleuropein, hydroxytyrosol, nutraceuticals

## Abstract

The current therapeutic scenario for inflammatory bowel diseases (IBD) involves aminosalicylates, corticosteroids, and immunomodulators, but concerns regarding their safety profiles and high costs heavily impact their widespread use. In recent years, the beneficial effects thatbiophenols—from fruit and vegetables—have on human health have been investigated. The antioxidant and anti-inflammatory properties of phenolic fraction, from olive leaves and fruits, have been suggested, and a potential application in gut inflammation has been supported by in vitro and IBD-animal models studies. In the present review, we first introduced the potential therapeutic role of olive tree biophenolsin chronic inflammatory disease. Then, we aimed to describe their most interesting application for gut inflammation, as the results of basic science studies and animal experimental models. Finally, the potential role of olive tree biophenols in the setting of human IBD is discussed.

## 1. Introduction

Inflammatory bowel disease (IBD) is a group of long-lasting idiopathic disorders characterized by a chronic inflammatory process involving the gastrointestinal (GI) tract. Both immunological dysregulation and environmental factors are key players in the pathogenesis of the disease. The main types of IBD are represented by ulcerative colitis (UC), affecting the large intestine (colon), and Crohn’s disease (CD), which can affect any section of the GI tract, while atypical forms are recognized as collagenous colitis and indeterminate colitis [1]. The signs and symptoms comprise abdominal pain, diarrhea, rectal bleeding, weight loss, fever, and fatigue, while stricture formation, abscesses, and fistulas are common complications [2]. Extra-intestinal manifestations, such as osteoarticular and skin involvement, can accompany the course of IBD, and are often the first evidence of the disease [3]. Furthermore, IBD patients are at an increased risk for colorectal cancer, mainly depending on the duration and extent of the disease [4]. Epidemiological data estimate that more than one million people in the United States and two million people in Europe suffer from IBD [5]. Aminosalicylates, corticosteroids, antibiotics, immunomodulators, and biological drugs represent the large variety of medical therapy currently available for the management of IBD. Nevertheless, limited efficacy and undesirable side effects heavily restrict these treatment strategies [6]. In the last decades, research efforts in the IBD field have been oriented towards complementary and alternative medicines, and several nutraceutical compounds have showed promising results in modulating intestinal inflammation while improving symptoms [7].

**Figure 1:**
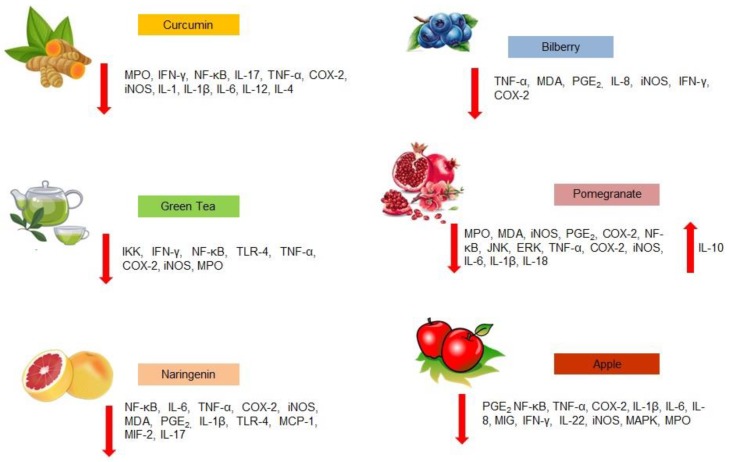
Molecular mechanisms of most investigated vegetable-derived biophenols (other than olive-derived) potentially involved in inflammatory bowel disease treatment. The red arrows pointing up indicate an increase while the red arrows pointing down indicate a decrease. MPO: myeloperoxidase; IFN-γ: interferon-gamma; NF-κB: nuclear factor kappa-light-chain-enhancer of activated B cells; MAD: malondialdehyde; PGE_2_: Prostaglandin E2; TLR-4: toll-like receptor-4; IL: interleukin; TNF-α: tumor necrosis factor-alpha; COX-2: cyclooxygenase-2; IKK: I kappa B kinase; iNOS: inducible nitric oxide synthase; JNK: c-Jun N-terminal kinase; ERK: extracellular signal regulated kinases; MIF-2: macrophage migration inhibitory factor-2; MCP-1: monocyte chemoattractant protein-1; MAPK: mitogen-activated protein kinase; MIG: monokine induced by gamma interferon.

Biophenols from fruit and vegetables are considered to be the most abundant source of antioxidants in the human diet. Green tea, pomegranate and apple biophenols, curcumin, bilberry anthocyanin, and naringenin showed interesting therapeutic effects for IBD, acting through the down-regulation of inflammatory pathways and enhancing antioxidant defenses (Figure 1) [8,9,10]. Accordingly, the great number of phenolic compounds present in olive tree (*Olea europaea* L.) represent the basis for their recognized beneficial effects on human health [11]. Antioxidant, anti-inflammatory, cardioprotective, hypocholesterolemic, and hypoglycemic properties exerted by olive tree biophenols have been demonstrated in humans and in animal models and, recently, phenolic fraction from olive leaves and fruits showed their potential application in gut inflammatory processes [12]. After introducing the relationship between olive tree-derived biophenols and inflammation, this review aims to explore the performance of phenolic fraction from olive trees in intestinal inflammation, focusing on the most studied compounds and describing the benefits that could derive from their use in the setting of IBD. 

## 2. Biophenols from Olive Tree and Inflammatory Diseases

**Figure 2:**
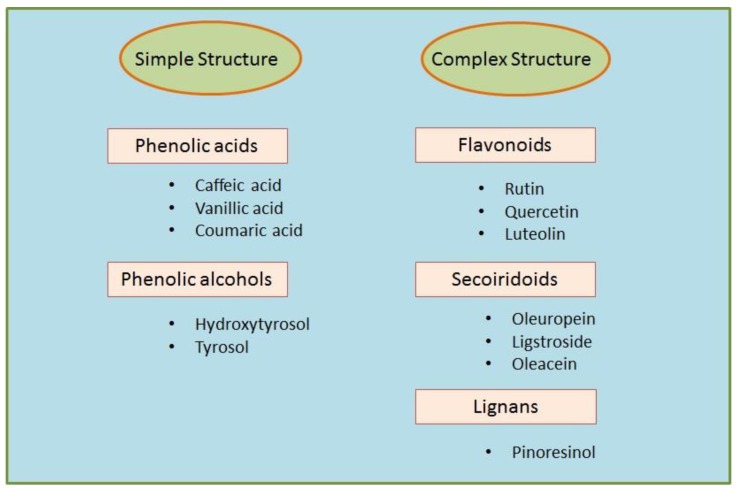
Classification of olive tree biophenols and most representative compounds.

**Figure 3:**
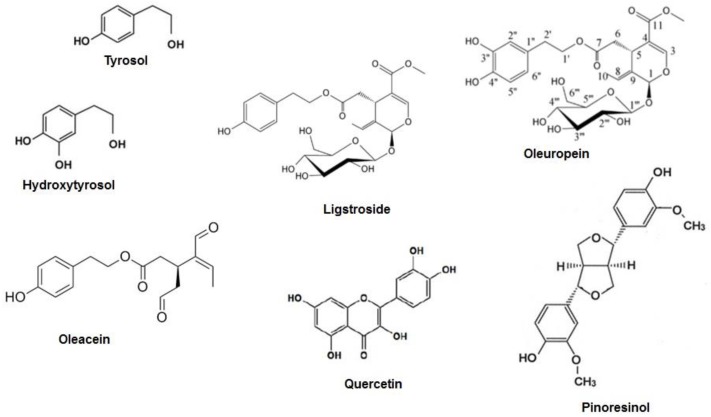
The chemical structure of the main phenolic compounds derived from olive tree (adapted from [18]).

**Figure 4:**
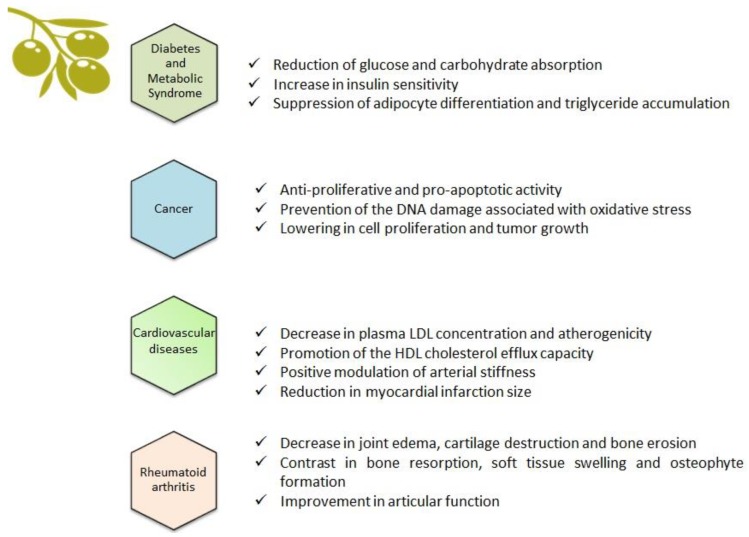
Chronic inflammatory diseases in which olive biophenols showed beneficial properties.

The main product obtained from the olive tree is olive oil, which comes from the fruit and accounts for the majority of the antioxidant activity attributed to the Mediterranean diet [13]. Olive leaves are considered to be a mixture of leaves and branches, derived from the pruning of the trees and the cleaning and harvesting process of the fruit [14]. As well as the fruit, olive leaves contain an abundance of biophenols, which can be extracted using special processing techniques [15]. This means that consumers can have access to one of the most beneficial components of olive oil without the necessity of consuming excessive amounts of it, therefore limiting the caloric intake which is still contained in the oil [16]. Among the biophenols of the olive tree, there are molecules with both simple structures, such as phenolic acids and phenolic alcohols, and complex structures, such as flavonoids, secoiridoids, and lignans (Figure 2 [17] and Figure 3 [18]). Oleuropein—which is mainly responsible for the bitter taste of olive fruits—and ligstroside are the most abundant secoiridoid compounds in green olive drupes [19]. The modulation of inflammatory pathways through the reduction of interleukin (IL)-6, cyclooxygenase (COX)-2, and metalloprotease has been suggested as an important mechanism underlying the beneficial effects of olive oil phenols [20]. In monocytes and monocyte-derived macrophages from healthy volunteers, the phenolic fraction of extra virgin olive oil (EVOO) lowered the expression of inducible nitric oxide (iNOS), peroxisome proliferator-activated receptor gamma (PPAR)-γ, and Toll-like receptor (TLR)-4 [21]. Oleuropein accounts for the major anti-inflammatory activity of olive leaf extract and, along with its derivative hydroxytyrosol, is the most extensively studied compound [22]. Post-traumatic inflammation in rats with spinal cord injuries is positively affected by treatment with oleuropein, which reduced the expression of tumor necrosis factor (TNF)-α, IL-1β, iNOS, and COX-2 [23]. Recently, the gastroprotective action of oleuropein has been demonstrated in an indomethacin-induced gastric ulcer model [24]. Hydroxytyrosol has shown a relevant anti-inflammatory action both in vitro—through the attenuation of pro-inflammatory agents iNOS, COX-2, and TNF-α in lipopolysaccharide (LPS)-challenged human monocytic Tamm-Horsfall protein (THP)-1 cells—and in animal models of inflammation, where reduced TNF-α and IL-1β expression has been found [25]. Oleocanthal, a secoiridoid derived from ligstroside and responsible for the burning sensation perceived at pharynx level after EVOO ingestion, demonstrated an ibuprofen-like anti-inflammatory profile thanks to its ability to inhibit COX-1 and COX-2 [26]. Collectively, data indicate that the nutraceutical properties of olive biophenols are strongly focused towards an anti-inflammatory profile, suggesting that a wide range of chronic inflammatory diseases could benefit from these biologically active compounds [27]; Figure 4. 

## 3. Diabetes and Metabolic Syndrome

Central obesity and insulin resistance are recognized as common underlying factors of diabetes and metabolic syndrome, while also being associated with a chronic inflammatory state [28]. At a molecular level, key biophenols from olive drupes and leaves, such as oleuropein and hydroxytyrosol, revealed the ability to modulate inflammation and cytokine-induced oxidative damage, lowering glucose and carbohydrate absorption, and increasing insulin sensitivity and related gene expression. They therefore suggest promising results in the prevention and management of type 2 diabetes [29]. In vitro, adipocyte differentiation and triglyceride accumulation are suppressed by oleuropein and hydroxytyrosol in a dose-dependent manner [30]. In mice, the protective effects of oleacein, one of the most abundant secoiridoids in EVOO, has been documented. After 5 weeks of treatment with 20 mg/kg of oleacein, animals showed significant improvement in insulin-dependent glucose and lipid metabolism. Furthermore, while liver histology was altered in mice fed with a high fat diet alone, when the animals received oleacein it remained similar to controls, fed with a normocaloric diet [31]. Regarding human studies, in healthy volunteers who consumed an olive leaf extract containing oleuropein and hydroxytyrosol, an improvement in vascular function and a reduction in inflammatory cytokines has been highlighted [32]. Accordingly, in a double blind randomized placebo-controlled trial, supplementation with 51.1 mg oleuropein and 9.7 mg hydroxytyrosol per day improved insulin sensitivity in middle-aged overweight men [33].

## 4. Cancer

A growing body of evidence is expanding the concept that chronic inflammation exerts a critical role in tumor development and progression [34]. Several studies carried out in cancer cell models have demonstrated the anti-proliferative and pro-apoptotic activity of olive biophenols, the underlying mechanisms of which are different according to cell type [35,36]. Olive leaf phenolic extract induced apoptosis in human colon cancer cells through the activation of caspase-3, -7, and -9, the promotion of reactive oxygen species (ROS)-induced endoplasmic reticulum stress, and an increase in intracellular Ca^2+^ concentration, which leads to mitochondrial dysfunction [37]. The potential antitumor activity of pinoresinol has been investigated in breast cancer cells and results support its cytotoxic, anti-proliferative properties, along with the prevention of the DNA damage associated with oxidative stress [38]. In tumor colon cell lines, hydroxytyrosol reduced cell proliferation and tumor growth through the inhibition of epidermal growth factor receptor expression [39]. Similarly, olive phenolic extract showed anti-proliferative activity in bladder cancer cells, and also a synergic effect when used in combination with the antineoplastic drugs mitomycin and paclitaxel [40]. The anti-cancer properties of secoiridoid fraction from olive trees have been documented in both animal models of cancer and human studies, confirming its ability to contrast the carcinogenesis process at both initiation and promotion/progression phases, and its protection toward DNA damage [41].

## 5. Cardiovascular Disease

Consumption of high-polyphenol-content olive oil decreased both plasma low density lipoprotein (LDL) concentration and atherogenicity in healthy volunteers, while promoting the high density lipoprotein (HDL) cholesterol efflux capacity which represents the main antiatherogenic function [42,43]. Olive leaf extract, containing oleuropein, hydroxytyrosol, verbascoside, luteolin, and quercetin, showed a protective ability in a model of oxidative damage of rat cardiomyocytes [44]. In a myocardial ischemia/reperfusion animal model, myocardial infarction size was reduced in rats treated with oleuropein [45]. The suppression of iNOS production seems to be the mechanism by which oleuropein protects against chronic doxorubicin-induced cardiomyopathy [46]. Two hundred healthy volunteers underwent plasma lipid levels and oxidative damage assessment after a daily administration of olive oil with high phenolic content, in a 3 week randomized crossover controlled trial [47]. Subsequent randomized controlled trials highlighted the hypotensive and lipid-lowering effects of phenolic-rich olive leaf extract in humans, particularly thanks to the secoiridoid fraction, which was able to positively modulate arterial stiffness and the vascular inflammatory profile [32,48]. The beneficial effects of olive tree extracts have been investigated also in thrombosis-associated factors such as platelet aggregation, fibrinolysis, and hemostasis, which are closely related to cardiovascular disease [49]. The CORonary Diet Intervention with Olive oil and cardiovascular PREVention study (CORDIOPREV study) confirmed the positive involvement of an EVOO-rich diet in influencing the incidences of cardiovascular events in subjects with documented coronary heart disease, during a 7 year observational period [50].

## 6. Rheumatoid Arthritis

A positive association between EVOO phenolic fraction consumption and an improvement in rheumatoid arthritis (RA) has been largely observed in mice. In experimental models of RA, animals showed a reduction in joint edema, cartilage destruction, and bone erosion, along with diminished levels of proinflammatory cytokines, prostaglandin E2, and COX-2 expression in the joint after dietary treatment with EVOO-polyphenol extract [51]. Similarly, hydroxytyrosol-supplemented refined olive oil determined a significant decrease in paw edema, bone resorption, soft tissue swelling, and osteophyte formation, improving articular function in treated animals [52]. In the same animal model of collagen-induced arthritis, hydroxytyrosol supplementation in the diet significantly prevented arthritis development, reducing cartilage matrix protein, metalloproteinase-3 levels, and levels of pro-inflammatory cytokines TNF-α, IFN-γ, IL-1β, IL-6, and IL-17A [53]. A topical formulation of hydroxytyrosol has been successfully used in Freund’s adjuvant-induced polyarthritic rats, which showed lowered arthritic scores, paw and ankle circumference, and serum IL-6 level after transdermal application of the compound [54]. In vitro studies support the anti-arthritic effects of phenolic extract from olive leaves, particularly oleuropein, on synovial fibroblasts, whose strong hyperproliferation in RA is responsible for the production of inflammatory mediators, followed by progressive matrix degradation, destruction of cartilage, and bone erosion [55,56]. In humans, a dry olive leaf has been administered orally in RA patients in addition to standard methotrexate therapy, in a trial that considers long term patients and early stage patients, compared with controls receiving methotrexate alone [57]. Interestingly, patients at the early stage of disease showed diminished levels of pro-inflammatory IL-6 and DNA damage, and a restored oxidative balance, suggesting a potential modulating role in the early phase of the disease. A randomized controlled 16 week clinical trial investigated the additional benefit of a formulation containing olive oil, figs, and olives in RA patients treated with disease-modifying antirheumatic drugs (DMARDs). This showed a trend of improvement in patient global assessment in the intervention group compared with the DMARDs alone group [58], although it was not significant—perhaps due to the short treatment period.

## 7. Biophenols from Olive Tree and Intestinal Inflammation

### 7.1. Basic Science Studies

At a molecular level, it has been indicated that EVOO biophenols are capable of modulating the oxidative, inflammatory, and immune status in the intestinal epithelial layer [59]. Recent findings in the cellular model of intestinal inflammation—obtained using human intestinal cell (Caco-2) lines—showed that polyphenol-rich olive oil supplementation significantly reduced the secretion of the main pro-inflammatory cytokine, IL-8, in both the basal and inflamed condition [60]. In the same cell type, the pro-oxidant and pro-inflammatory effects of a mixture of dietary oxysterols have been counteracted by pre-treatment with an olive-derived phenolic extract, in part through a modulation of the NF-κB activation pathway [61]. Hydroxytyrosol revealed the ability to diminish the acrylamide-induced oxidative stress in Caco-2 cells by reducing ROS generation, recovering the antioxidant defenses and decreasing caspase-3 activity [62]. Recently, the phenolic fraction derived from a particular Italian cultivar of olives was found to be involved in restoring the loss of the epithelial integrity and repairing the membrane oxidative damage after induced peroxidative stress in enterocyte-like cells [63]. While looking for the molecular mechanisms involved in these promising anti-inflammatory activities, Muto E et al. demonstrated that EVOO phenolic extract inhibits the LPS-induced IL-8 secretion, by interference with NF-κB signaling and p38 MAPK pathway in Caco-2 cells [64]. Authors demonstrated that, besides regulating gene transcription, olive oil phenols affected mRNA stability, therefore suggesting that olive-derived biophenols may act at both transcriptional and posttranscriptional levels in intestinal epithelial cells.

Recently, oleuropein has been studied in human colon biopsies, taken from UC patients and grown according to an ex vivo organ culture model. In both protein extracts and supernatants from colonic biopsies, which were challenged with oleuropein, a significant reduction in IL-17 levels—which has been found to be increased in the colonic mucosa and serum of UC patients—has been documented. Furthermore, the treatment of biopsy samples with oleuropein led to a restoration of the typical microscopic damage of UC, with a strong decrease in the inflammatory infiltrate, disappearance of focal cryptitis/crypt abscesses, and recovery of mucin-forming goblet cells. These results were likely obtained through the modulation of the CD3+ and CD4+ subsets of T lymphocyte cells, whose number dramatically decreased at the immunohistochemistry assessment in samples challenged with oleuropein [65].

Another interesting study evaluated the immunomodulatory properties of an olive leaf extract, containing more than 80% oleuropein, in ex vivo organ cultures of mucosal explants from CD patients [66]. Authors measured proinflammatory cytokine expression after 24 h of treatment, reporting the reduction in IL-1β, IL-6, and IL-8 mRNA levels and therefore supporting the involvement of olive leaf extract in ameliorating the intestinal inflammatory milieu.

### 7.2. Results from Animal Models of IBD

In vivo studies using animals with experimentally induced colitis indicate that EVOO-derived phenolic compounds can be effective in preventing and treating intestinal inflammation and related injury [67]. In an experimental model of acetic acid-induced colitis in rats, dry olive leaf extract was administered orally for two consecutive days, starting from the day of colitis induction [68]. Macro- and microscopic evaluation of the colon showed an olive leaf extract dose-dependent attenuation of inflammation, as demonstrated both by the reduction of ulcerative lesions and the decrease in TNF-α and IL-2 levels in colonic homogenates.

Giner et al. investigated the anti-inflammatory effect of oleuropein in a dextran sodium sulfate (DSS)-induced colitis mouse model [69]. After the sacrifice of the animals, an examination of the colons from mice fed with oleuropein showed a reduced colon shortening and, at the histological assessment, the colon tissue exhibited a significantly lower degree of mucosal injury, as supported by fewer infiltrating cells and less edema. Furthermore, the activity of the myeloperoxidase (MPO) enzyme, produced by polymorphonuclear leukocytes and acting as a marker of neutrophil infiltration, was dramatically reduced in the colon tissue from oleuropein-treated mice, as well as IL-1β, IL-6, TNF-α, and COX-2 production. Subsequent research from the same group suggests another role for oleuropein in protecting against chronic inflammation, since colon tissue from treated mice showed a reduced neutrophil, macrophage, and eosinophil accumulation, and an increased IL-10 production, which is known to act as an anti-inflammatory cytokine [70]. Interestingly, the modulatory effect of oleuropein has been documented on the T helper (Th)17 response in DSS acute colitis in mice [71]. Indeed, in colonic samples from treated animals, oleuropein inhibited Th17 response, by decreasing CD4+Rorγt+, IL-17+, IFN-γ+ T-cell subsets in the lamina propria, as well as IL-17A expression. Dietary supplementation with polyphenol-enriched EVOO was found to protect against organ damage, through proliferator-activated receptor gamma (PPARγ) up-regulation and mitogen-activated protein kinases (MAPKs) inhibition [72]. Authors used the above-mentioned mouse model of DSS-induced chronic colitis and fed animals with a peculiar phenolic extract from olive oil, mainly containing hydroxytyrosol, oleuropein, pinoresinol, and ligstroside, thus confirming its ability to modulate the inflammatory cascade of these compounds. Recently, topical administration of an aqueous solution containing hydroxytyrosol provided the first evidence that an intrarectal administration of hydroxytyrosol reduces the severity of the inflammatory damage during experimental colitis. It is worth noting that the compound proved to be safe, since treatment did not show any adverse effect in the animals [73]. 

**Figure 5:**
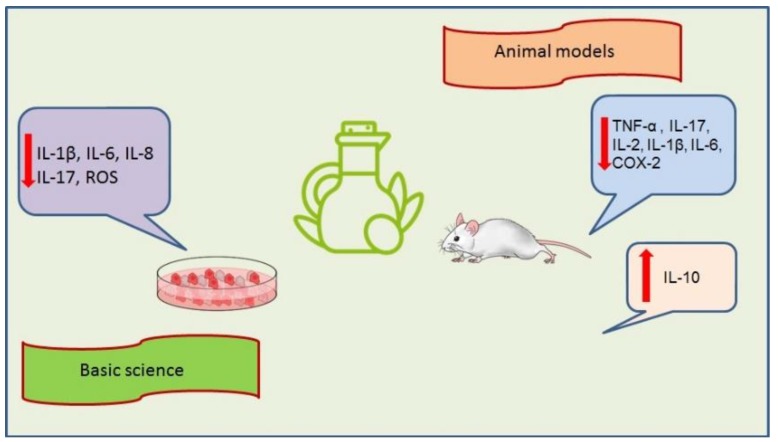
Biophenols from olive tree and intestinal inflammation: results from basic science and animal models. The red arrows pointing up indicate an increase while the red arrows pointing down indicate a decrease.

Effects of olive tree-derived biophenols from basic science and animal models studies are summarized in Figure 5.

### 7.3. Potential Role in Human IBD

Olive oil is one of the cornerstones of the Mediterranean diet, whose benefits for human health are well recognized [74]. Dietary habits seem to be potentially associated with IBD [75]. Moreover, adherence to a Mediterranean diet could influence the pathogenesis and course of the disease [76]. IBD appears to be disorders resulting from dysregulation between host genetics, immune system, and gut microbiota [77]. It has been widely documented that EVOO is capable of exerting a targeted modulation on gut microbiota in comparison to other fats [78,79], and the consequent multiple benefits of this positive impact on intestinal microbiota have been described in mice [80,81]. In humans, it has been demonstrated that a Mediterranean diet promotes healthy intestinal microbiota and protects from bowel alterations [82]. Gut microbial composition is strongly affected by EVOO biophenols and this interaction could explain the prominent role of the Mediterranean diet in gut health homeostasis [83]. In a randomized controlled double blind crossover human trial, the benefits of olive oil phenolic compounds have been investigated with regards to intestinal immunity [84]. Fecal immunoglobulin A (IgA) and IgA-coated bacteria increased after three weeks of the ingestion of 25 mL/day of olive oil, thus confirming its ability to stimulate the immune system in humans.

Another randomized controlled double blind crossover human trial evaluated fecal quantitative changes in microbial population after three weeks ingestion of 25 mL/day of phenolic-enriched olive oil, showing both the fecal increase in bifidobacteria and the fecal hydroxytyrosol and dihydroxyphenylacetic acids, which are microbial metabolites of the phenolic compounds responsible for antioxidant activity [85]. As can be seen—notwithstanding the promising results obtained from in vivo and animal models of colitis which suggest the benefits of EVOO in chronic inflammatory bowel conditions—there are few studies that focus on humans. Moreover, investigations are needed to characterize the pharmacokinetic and pharmacodynamics properties of EVOO biophenols in human beings, and to set up large, multicenter, clinical trials to ensure the quality of the evidence obtained [86].

## 8. Conclusions

The therapeutic potential of biophenols derived from the olive tree in human IBD is an intriguing topic, but their actual contribution to inducing and maintaining remission needs to be elucidated by well-designed randomized clinical trials. At the same time, further research is needed to clarify the mechanisms underlying the beneficial effects from the phenolic fraction of olive trees, in order to design appropriate therapeutic strategies and recommendations.
